# Florid Cemento-Osseous Dysplasia-Associated Simple Bone Cyst Showing Marked Irregular Border and High Apparent Diffusion Coefficient Value

**DOI:** 10.1155/2020/8854428

**Published:** 2020-09-21

**Authors:** Ikuho Kojima, Takashi Nishioka, Maya Sakamoto, Yuko Sai, Yushi Ezoe, Masahiro Iikubo, Hiroyuki Kumamoto, Tetsu Takahashi

**Affiliations:** ^1^Division of Oral Diagnosis, Tohoku University Graduate School of Dentistry, 4-1 Seiryo-machi, Aoba-ku, Sendai, Miyagi 980-8575, Japan; ^2^Division of Oral and Maxillofacial Surgery, Tohoku University Graduate School of Dentistry, 4-1 Seiryo-machi, Aoba-ku, Sendai, Miyagi 980-8575, Japan; ^3^Division of Oral Pathology, Tohoku University Graduate School of Dentistry, 4-1 Seiryo-machi, Aoba-ku, Sendai, Miyagi 980-8575, Japan

## Abstract

A simple bone cyst (SBC) is an intrabone cavity without epithelial lining, which occasionally occur with fibrous-osseous lesions. The imaging finding of the scalloped border and conserving the lamina dura, periodontal ligament spaces, or follicular spaces are considered radiographically pathognomonic of SBC. SBC has been reported to occur with fibrous-osseous lesions, including cemento-osseous dysplasia (COD). Computed tomography (CT) imaging findings are sparse, and there are no reports of magnetic resonance imaging (MRI) findings, including apparent diffusion coefficient (ADC) value calculated by diffusion-weighted MRI for the florid COD-associated SBC. We report a case of a 39-year-old woman who was referred to our hospital because a panoramic radiograph of her right mandible showed asymptomatic radiolucency in the apical molar area. CT images showed a low-density lesion in the periapical areas of the right mandible, which accompanied a well-defined, high-density lesion, and a mixed low/high-density lesion with an unusual marked irregular border in the left mandible. The MRI showed cyst-like extremely hyperintense signals on fat-suppressed T2-weighted images at the right mandibular low-density and the left mandibular mixed low/high-density areas. ADC map showed high values (over 2.5 × 10^−3^ mm^2^/s) at the cystic areas in the bilateral mandible. We performed incisional biopsies of the bilateral mandibular lesions and resections of the bilateral maxillary lesions. Surgical and histopathological findings established a diagnosis of florid COD in the bilateral mandible and maxilla, simultaneously complicated by multiple SBCs in the bilateral mandible. The ADC value of cystic component is useful for the diagnosis, if the COD-associated SBC exhibits solitary, nonspecific, or rare imaging findings.

## 1. Introduction

A simple bone cyst (SBC) is a pseudocyst, which is diagnosed based on the finding of an intrabone cavity without epithelial lining [1]. SBC appears as a well-defined cystic lesion with thin sclerotic borders or an ill-defined cystic lesion on the panoramic radiograph. In the jaws, the imaging appearances of the scalloped border and conserving the lamina dura, periodontal ligament spaces, or follicular spaces are considered radiographically pathognomonic of SBC [2]. SBCs are often found incidentally in young patients and resolve spontaneously after aspiration of the cavity or surgical biopsy without curettage [1, 3]. Interestingly, SBC has been reported to occur with fibrous-osseous lesions, including cemento-osseous dysplasia (COD) [4–8]. Although the radiologic features of SBC or COD, including computed tomography (CT) imaging findings, are well described, those of florid COD-associated SBC are sparse. Moreover, differentiation of SBC from other cystic lesions may be difficult when these lesions appear with nonspecific cystic or unusual imaging appearance. Magnetic resonance imaging (MRI) features of SBC are previously reported using apparent diffusion coefficient (ADC) calculated by diffusion-weighted MRI (DW-MRI) [9–11]. However, there are no reports of MRI findings for COD-associated multiple SBCs. The differential diagnosis is difficult, if the COD-associated SBC exhibits solitary, nonspecific, or rare imaging findings. The purpose of this report was to describe the findings of a panoramic radiograph, CT, MRI, and bone scintigram of florid COD-associated SBCs adjacent to radiopaque bone/cementum-density masses in multiquadrant periapical regions.

## 2. Case Presentation

This case report was approved by our institutional ethics committee (No. 18651). All procedures followed were in accordance with the ethical standards of the responsible committee on human experimentation (institutional and national) and with the Helsinki Declaration of 1975, as revised in 2008. Informed consent was obtained from the patient for being included in the study.

### 2.1. Patient

A 39-year-old woman had asymptomatic radiolucency of the right mandible on X-ray radiographic examination for caries treatment at a primary dental office. She was referred to our hospital for further radiographic examination and treatment of the lesion. Her medical history revealed postoperative uterine cervical cancer. She had no significant family history. Clinical findings of extra- and intraoral appearances were unremarkable.

### 2.2. Imaging Findings

**Figure 1:**
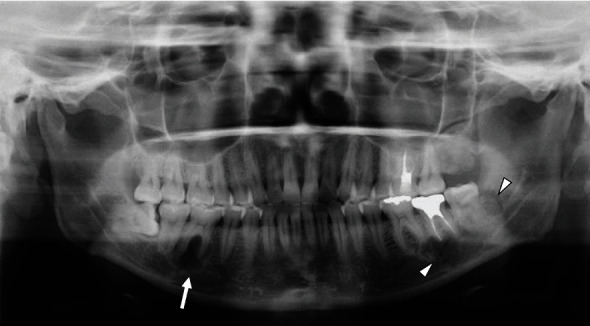
Panoramic X-ray radiogram. Radiolucent lesions are seen in the right mandible (*arrow*). The high-density lesion is complicated by the periapical right mandibular 2nd molar. Additionally, mixed radiolucent/radiopaque lesion was shown in the left molar region (*arrowheads*).

**Figure 2:**
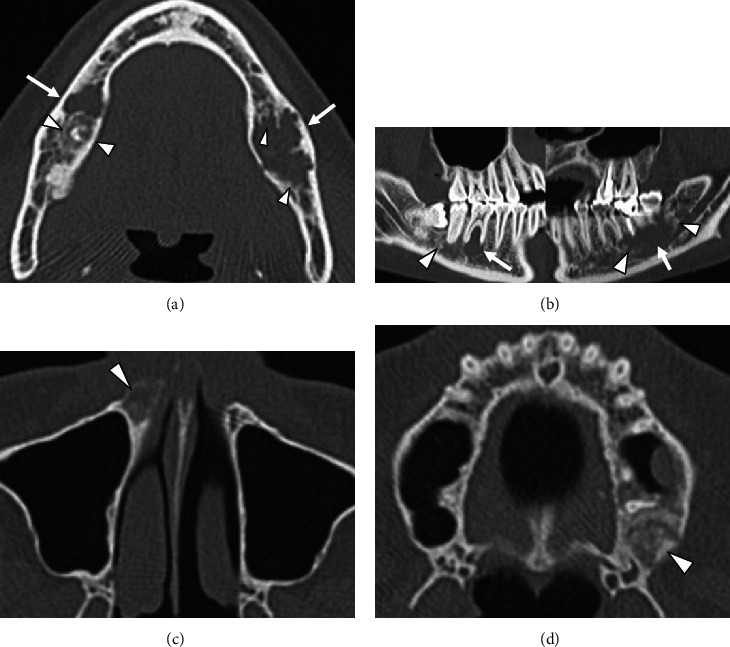
CT images. (a, b) Well-defined, low-density lesions (*arrows*) with scalloped border, which accompanies a well-defined high-density appearance (*arrowheads*) are observed in the right mandibular molars on transverse and sagittal images. The left lesion shows mixed main low- (*arrows*) and partial high- (*arrowheads*) density areas with an unusual, marked irregular border in the molar region. The right lesion shows a unilocular, slightly irregular border. (c, d) Transverse CT images show high-density lesions in the right anterior and left posterior maxilla (*arrowheads*). CT: computed tomography.

**Figure 3:**
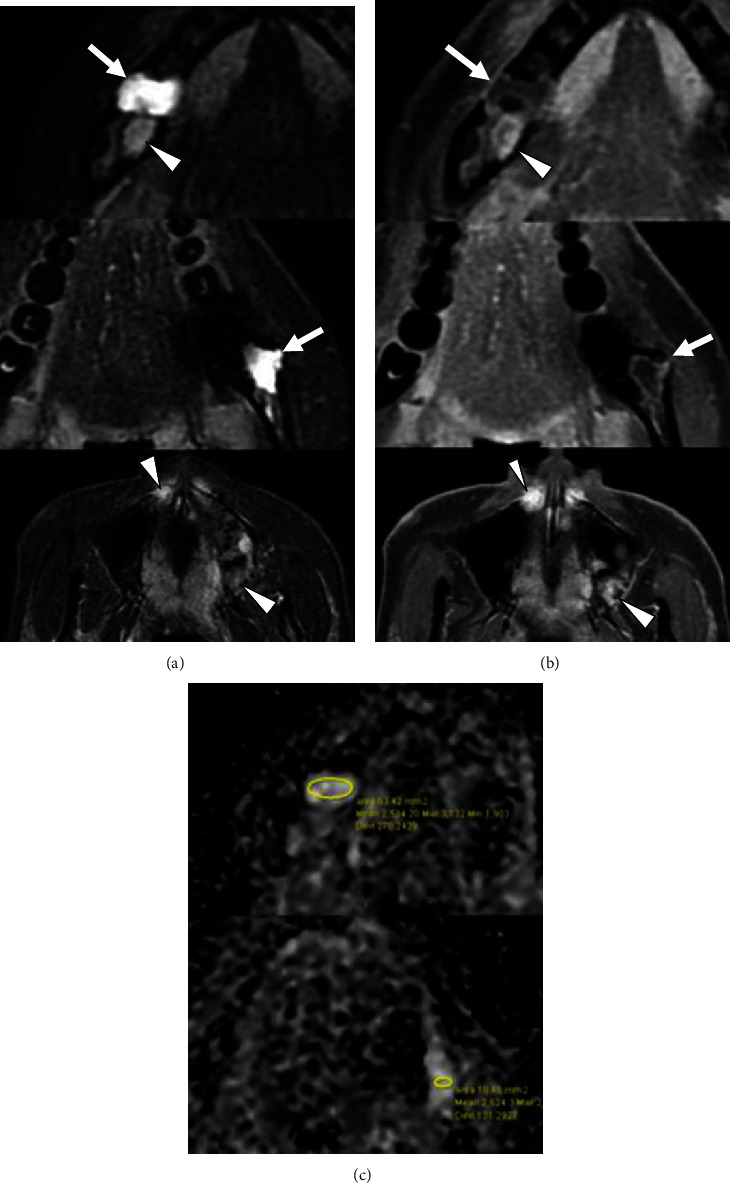
Magnetic resonance images. (a) Transverse fat-suppressed T2-weighted images show extremely hyperintense signal areas in the bilateral mandible (*arrows*) and hyperintense signal areas in the right mandible and bilateral maxilla (*arrowheads*). (b) Contrast-enhanced T1-weighted images show thin, peripherally enhanced lesions in the bilateral mandible (*arrows*) and heterogeneously enhanced lesions in the right mandible and bilateral maxilla (*arrowheads*). (c) The ADC maps show extremely high ADC values (over 2.5 × 10^−3^ mm^2^/s) at cystic areas in the bilateral mandible. ADC: apparent diffusion coefficient.

**Figure 4:**
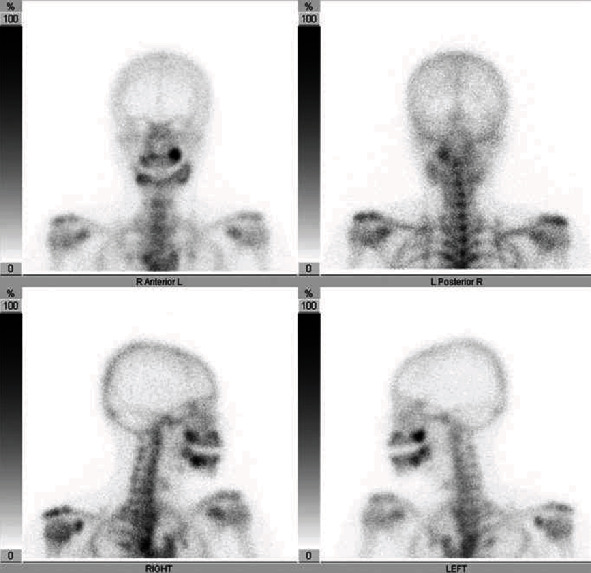
Bone scintigram. Tracer accumulations are observed in the high-density lesion on CT finding of the mandible and maxilla.

Panoramic X-ray radiograph showed radiolucency in the apical region of the vital right lower 1st molar. Mixed radiolucent/radiopaque lesion was shown in the left molar region (Figure 1). CT findings showed multiple low- or high-density lesions in the bilateral mandible and additional high-density lesions in the bilateral maxilla. The right mandibular lesion showed a well-defined low-density lesion with scalloped border in the periapical areas of the right lower 1st molar, which accompanied a well-defined high-density lesion in the adjacent 2nd molar periapical area (Figures 2(a) and 2(b)). The left lesion presented mixed low/high-density lesion with an unusual, marked irregular border that was more irregular than the scalloped border in the molar region (Figures 2(a) and 2(b)). Both these lesions showed erosion of the cortical bone. Additional high-density lesions were observed in the left 3rd molar and right lateral incisor areas of the maxilla, which were well-defined (Figures 2(c) and 2(d)). No demonstrable finding of root resorption was detected on CT. The MRI showed cyst-like extremely hyperintense signals on fat-suppressed T2-weighted images and thin peripheral enhancement on contrast-enhanced T1-weighted images at the right low-density areas (Figures 3(a) and 3(b)). Hyperintense signals on fat-suppressed T2-weighted image and heterogeneous enhancement on contrast-enhanced T1-weighted image were shown in the adjacent 2nd molar peripheral area of the right high-density lesion (Figures 3(a) and 3(b)). For left mandibular mixed low/high-density lesion on CT finding, MRI showed marked irregular cystic/fluid-collected lesion that was hyperintense signals on fat-suppressed T2-weighted image and thin peripheral enhancement on contrast-enhanced T1-weighted image (Figures 3(a) and 3(b)). No demonstrable findings were found about the high-density area in the left mandible, which may be due to metal artifact on MRI. Although no hyperintense signals were found on DW-MRI, ADC showed high values (over 2.5 × 10^−3^ mm^2^/s) at cystic areas in the bilateral mandible (Figure 3(c)). Compared to the CT findings, bone scintigram results showed high tracer uptake in the high-density lesions (Figure 4).

### 2.3. Clinical Diagnosis

We diagnosed this case as a florid COD-associated SBC based on the plural cystic lesions showing high ADC value with multiquadrant fibrous lesions.

### 2.4. Treatment and Prognosis

**Figure 5:**
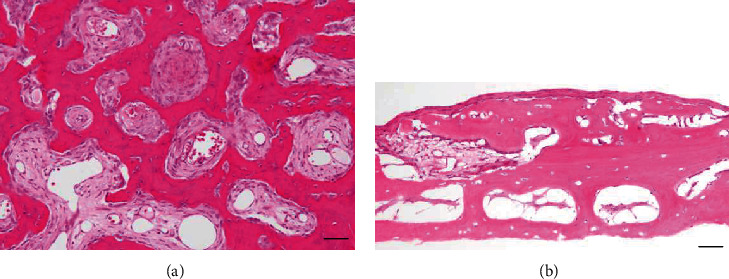
Histopathological findings (original magnification, ×100). (a) Specimens of the high-density lesions comprised irregular piece of fibrous connective tissue adjacent to the woven bone trabecula, which had an osteoid rim-like border and prominent osteoblasts. Bar: 100 *μ*m. (b) Specimens of the bilateral mandibular low-density lesions exhibited remnants of a partial bony cavity lined by slightly compressed connective tissue. Bar: 100 *μ*m.

Incisional biopsies of the bilateral mandibular lesions and resection of the bilateral maxillary lesions were performed. Gross examination revealed a mixture of tiny bone-like material within an empty cavity bone in the bilateral mandible. The high-density lesions that resembled bone-like structures were easily removed. Histopathological examination of the tissue specimens was performed. The histopathological findings showed multiple irregular pieces of fibrous connective tissue adjacent to the woven bone trabeculae with an osteoid rim-like border and prominent osteoblasts (Figure 5(a)). Histological examination of the bilateral mandibular specimens of the peripheral low-density lesions showed remnants of a partial bony cavity lined by slightly thin connective tissue. There was no epithelium component in the tissue (Figure 5(b)). Both surgical and histopathological findings matched the diagnosis of a florid COD, which were simultaneously complicated by multiple SBCs.

**Figure 6:**
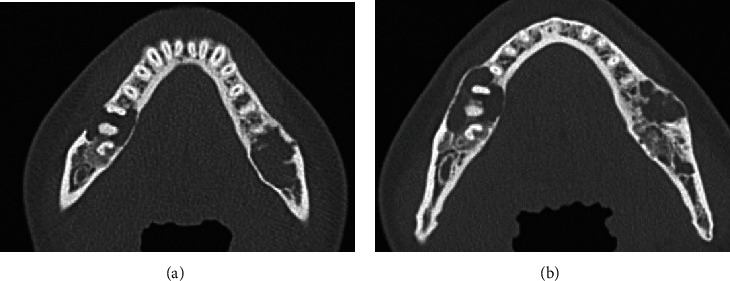
Follow-up CT images. (a) Three months and (b) twenty-seven months after the incisional biopsy; the CT images indicate enlargement of the right and an anterior half area of the left low-density lesions. CT: computed tomography.

According to the standard treatment protocol for each of the two lesions, we longitudinally followed up the mandibular lesions after the biopsy by routine imaging examination. A posterior half area in the mixed low/high-density lesion of the left mandible and resected cavities of the bilateral maxilla gradually decreased in size with bone formation. In contrast, twenty-seven months after the biopsy, a follow-up CT showed that an anterior half area in the left mandibular lesion enlarged and changed to multilocular shape. The unilocular, slightly irregular border, low-density lesion of the right mandible, also enlarged with bone expansion (Figure 6). Since then, we have continuously followed up this patient.

## 3. Discussion

The CT findings of our patient showed low-density lesions next to the high-density lesion of the periapical areas in the right mandibular molars and mixed low/high-density lesion in the left mandibular molars; these lesions had an irregular border, cortical bone erosion, and loss of the lamina dura. In particular, the appearance of a marked, irregular border in the left mandibular lesion on CT image was rare for the patient with SBC [2, 3]. If the left mandibular lesion presented solitary mixed low/high-density with an irregular border, desmoplastic ameloblastoma should be considered a first differential diagnosis. There are no reports of MRI findings for COD-associated multiple SBCs. We could diagnose this case as a florid COD-associated SBCs based on the plural high ADC cystic lesions, which suggested SBC with multiquadrant fibrous lesions. Eida et al. [10] reported the efficacy of the ADC value of the cystic component on DW-MRI for differentiation of odontogenic lesion and demonstrated SBC and ameloblastoma had a high ADC value. Desmoplastic ameloblastoma that presented mixed radiolucent-radiopaque appearance with ill-defined borders has been radiographically reported in many cases [12–14]. Although ameloblastoma showed high ADC value in previous reports [10, 11, 15, 16], there is no report on the ADC of the desmoplastic variant. Therefore, desmoplastic ameloblastoma might be considered a differential diagnosis, if a solitary lesion. The imaging finding of bone scintigraphy showed tracer accumulations in the high-density lesion of CT finding of the mandible and maxilla. This finding was nonspecific and consistent with previously published reports [17, 18]. In this report, we have presented the case of a patient with florid COD-associated SBCs diagnosed by the imaging finding of plural cystic lesion exhibiting high ADC value with multiquadrant high-density lesions.

Follow-up CT images obtained twenty-seven months after the incisional biopsy indicated that part of the lesions seen previously had gradual regression with bone formation. On the contrary, the bilateral SBCs showed enlargement in size and changed to marked multilocular low-density lesions with bone expansion after the biopsy. Suei et al. [3] retrospectively reviewed 108 cases of solitary SBC from the literature and thirty-one of their cases and demonstrated that the SBCs with multiple, scalloped borders, bone expansion, root resorption, a radiopaque mass including fibrous-osseous lesions, or loss of the lamina dura had a higher recurrence rate (enlargement after surgery including only curettage and exploration of the intrabone cavity wall) than the lesion with smooth borders, no bone expansion, and intact lamina dura. On the other hand, no histopathological analysis has been studied on the recurrence rate of SBCs. Matsumura et al. [19] reviewed the correlation between histopathological and radiological findings of fifty-three cases of SBCs and demonstrated that many SBCs radiographically presenting bone expansion and radiopaque finding had histopthologically thickened wall with dysplastic bone formation. Recurrent SBCs might have a varying degree of osteoblastic change irrespective of the presence or absence of the radiopaque masses. Taking these reports into account, therefore, the marked irregular border on CT images of our case may reflect heterogeneous fibrous-osseous changes in the lesion's peripheral margin. We cannot precisely analyze the histopathological finding, and no other demonstrable finding of MRI was found for the irregular border in the recurrence. In the future, histopathological analysis or MRI finding review of the irregular border might be expected to explain the mechanism of recurrence and contribute to treatment decisions.

Some previous reports assessed the subject population, age distribution, and anatomical distribution of patients with SBC. In one of the previous reports which had the largest number of cases, 87% (20/23) of COD-associated SBCs occurred in women with a mean age in the 40s, and the majority (95.7%) of the cases occurred in the mandibular molar area [8]. In contrast, solitary SBCs were found in equal numbers in both sexes in their second decade of life and typically occurred in the anterior mandible [5–8]. These differences may be related to the pathogenesis of the disease. Chadwick et al. [8] discussed that solitary and COD-associated SBCs might be the same lesion but arising as separate lesions owing to different biological circumstances such as differences in the mean age, sex ratio, and anatomical distribution. They hypothesized that in adolescents, disturbance of normal osteoblast and osteoclast activity may depend on a new and constantly changing biomechanical property of the mandible during growth and development. This indicates that bone cells, especially the osteoblasts, could not satisfy these demands. In contrast, in patients with full skeletal maturity, and especially in women with a significantly higher incidence of COD-associated SBCs, the occurrence of SBCs might indicate differential activity in the normal bone cells because of different underlying reasons. Bones of middle-aged women with possible osteoporosis are more likely to have low or insufficient osteoblast numbers. If this hypothesis is credible, COD-associated SBC caused by osteoporosis in middle-aged women is likely to have a high recurrence rate regardless of imaging findings. The histopathological analysis is expected as evidence.

## 4. Conclusion

We have reported an interesting case of florid COD-associated multiple SBCs, which showed a rare CT finding of mixed low/high-density with the marked irregular border and high ADC values of cystic components on MRI. The ADC value of cystic component is useful for the diagnosis, if the COD-associated SBC exhibits solitary, nonspecific, or rare imaging findings.
